# Declared Inactive Ingredients in US Oral Drug Listings: A Reproducible Census of Prevalence, Labeling Coverage, and Within-Name Variation

**DOI:** 10.7759/cureus.114947

**Published:** 2026-08-21

**Authors:** Brian Staiger

**Affiliations:** 1 Pharmacy, University at Buffalo, State University of New York, Buffalo, USA

**Keywords:** drug labeling, inactive ingredients, medication safety, national drug code, pharmaceutical excipients, structured product labeling

## Abstract

Background

A current assessment of structured drug labeling can update excipient-declaration prevalence, quantify structured-data coverage, and describe variation among listings sharing a prescription generic name. Building on prior formulation-level analyses, this study contributes a current, fully reproducible public-data snapshot with pharmacologic-class-resolved declaration patterns and a within-generic-name declaration-consistency analysis.

Methods

We conducted a cross-sectional analysis of a published census derived from the openFDA National Drug Code (NDC) Directory export dated 2026-07-18 and DailyMed Structured Product Labeling (SPL). The analytic denominator comprised 50,005 distinct oral Product NDC listings with an exact SPL product match and at least one structured inactive-ingredient (IACT) declaration. We described listing-level excipient burden and prevalence, unique qualitative excipient profiles, structured-declaration availability, and patterns across dosage forms, marketing categories, and Established Pharmacologic Classes (EPCs). A predefined Unique Ingredient Identifier (UNII)-based panel identified selected avoidance-relevant excipients. Among prescription generic-name groups with at least two listings, we identified affected groups in which every observed listing declared the target.

Results

Listings declared a median of nine excipients (interquartile range {IQR}, 6-12; maximum, 41). Magnesium stearate (68.07%), titanium dioxide (53.27%), and microcrystalline cellulose (52.88%) were most prevalent. The 50,005 listings contained 13,540 unique qualitative declared excipient profiles, a mean of 3.69 listings per profile. Among exact SPL product matches, 97.99% contained at least one structured IACT declaration. Lactose was declared in 39.34% of listings, gelatin in 18.5%, and at least one panel-defined synthetic color additive in 39.96%. Within affected prescription generic-name groups, every observed listing declared lactose in 306 of 657 groups (46.58%) and gelatin in 150 of 322 groups (46.58%).

Conclusions

Declared excipients were common and varied across product and pharmacologic-class strata. Many listings shared the same qualitative excipient profile, although profile identity does not establish formulation identity. In some prescription generic-name groups, every observed listing declared an avoidance target. These findings describe structured declarations rather than actual composition, dispensing exposure, or therapeutic interchangeability.

## Introduction

Oral drug products contain multiple inactive ingredients that serve manufacturing, stability, appearance, and palatability functions. Although most are tolerated by most patients, selected excipients matter to identifiable groups of people, and the reasons are clinically specific: lactose in lactose intolerance and galactosemia; gelatin, peanut oil, sesame oil, and soy-derived ingredients in IgE-mediated allergy; aspartame as a phenylalanine source in phenylketonuria; sorbitol and sucrose in hereditary fructose intolerance and in conditions where osmotic carbohydrate load matters; benzyl alcohol and propylene glycol in neonatal and pediatric exposure limits; and porcine- or bovine-derived gelatin in religious and dietary decision-making. The avoidance-relevant targets considered here include lactose, explicitly wheat-derived ingredients, peanut oil, sesame oil, soy-derived ingredients, aspartame, gelatin, and selected synthetic color additives [1].

The labeling layer through which these questions are answered has itself become clinically consequential. Patients, pharmacists, and prescribers increasingly resolve "does this product contain X?" through automated lookups against Structured Product Labeling (SPL) rather than by reading a package insert or telephoning a manufacturer. The coverage, granularity, and consistency of that structured layer therefore determine whether such a lookup returns a correct answer, an incomplete answer, or a silently wrong one. That property has not previously been quantified on current data.

Prior work established that inactive ingredients are widespread in US oral solid medications and that formulations of the same active ingredient can differ in their excipient composition. Reker et al. used Pillbox data to characterize ingredient burden, allergy-associated ingredients, unique ingredient combinations, and the availability of alternative formulations for active ingredients [1]. That study established the scale and clinical relevance of the issue. A current assessment using actively listed National Drug Code (NDC) records and structured DailyMed labeling can additionally address the coverage and limitations of public data now used in consumer and clinical drug-information tools.

Several questions therefore remain relevant, and each maps to a decision a clinician or patient actually makes. What do current US oral Product NDC listings declare, which is the denominator for any counter-side question about a specific product? How are declarations distributed across dosage forms, marketing categories, and pharmacologic classes, which indicate where an avoidance request is likely to be straightforward and where it is likely to require manufacturer contact? How often do identical qualitative excipient profiles recur across listings? How often do exactly matched SPL product records contain any structured inactive-ingredient declaration, which bounds how often an automated tool must return "unknown" rather than "not present"? Finally, within prescription generic-name groups, how often does every observed listing declare a selected avoidance target, which indicates when switching manufacturers within a name cannot resolve an avoidance request?

We addressed these questions using a published, reproducible census of declared inactive ingredients in the US oral drug-listing snapshot. The primary objective was to characterize excipient burden, prevalence, and structured-declaration availability. Secondary objectives were to describe co-occurrence and qualitative excipient-profile diversity, to characterize patterns for a predefined avoidance-relevant panel overall and by-product and Established Pharmacologic Class (EPC) strata, to quantify uniform target declaration within prescription generic-name groups and its sensitivity to group composition, and to make every reported figure reproducible from deposited artifacts. The analysis concerns structured declarations, not confirmed product composition, patient exposure, or therapeutic interchangeability.

## Materials and methods

Study design, setting, and data source

This was a descriptive cross-sectional analysis of the published dataset Declared Inactive Ingredients in US Oral Drug Product Listings: A Census of 51,065 Eligible Distinct Product NDC Listings (version 1.0), which the author also produced and deposited at Zenodo [2]. The study population was derived from the openFDA NDC Directory export dated 2026-07-18 [3]. The NDC Directory contains labeler-submitted drug-listing information; inclusion does not mean that the FDA has verified the entry, approved the product, or confirmed current retail availability.

Sequential eligibility criteria identified 51,065 distinct Product NDC listings that were finished human prescription or over-the-counter (OTC) drugs, were listed with an exclusively oral route, and had a mappable swallowed-oral dosage form. These listings spanned nine source dosage-form groups. DailyMed SPL XML was retrieved for all 36,325 associated SPL Set IDs; 22 retrieval failures affecting 32 listings were retained explicitly in the source census [4].

Inactive ingredients were extracted only from direct SPL ingredient nodes with classCode="IACT" and only when exactly one SPL product block matched the Product NDC listing. Ingredients were keyed by Unique Ingredient Identifier (UNII) where present. The source census reports 14 automated validation checks and 18 stratified, pipeline-independent manual XML spot checks; the present analysis reverified the downloaded files against the census's published SHA-256 checksums.

The study size was determined by the complete set of listings meeting the source census eligibility criteria in the dated export; no sample-size calculation or sampling was performed.

Analytic populations and missing structured declarations

The primary listing-level denominator comprised the 50,005 listings with an exact SPL product match and at least one structured IACT declaration (included_in_frequency_denominator). Listings without an exact match, without any structured declaration, or with retrieval failures were described in the cohort accounting but excluded from ingredient-frequency analyses. Missing declarations were treated as unknown and were not imputed as ingredient absence.

Denominators differed where specified. Structured-declaration availability used the 51,065 eligible listings and the subset with an exact SPL product match. EPC analyses used EPC-specific, overlapping denominators. The within-name analysis used prescription generic-name groups meeting its stated eligibility criteria. All other listing-level analyses used the 50,005-listing primary denominator.

Avoidance-relevant excipient panel

Each panel group was defined as an explicit UNII set drawn from the census ingredient key space, listed in full with its inclusion and exclusion rationale in the Appendices: lactose (three UNIIs), gelatin (eight), explicitly declared wheat (two: wheat starch and wheat germ), peanut oil (one), sesame oil (one), soy-derived ingredients (11), and synthetic color additives (32: certified FD&C/D&C colorants, including carbon-black D&C Black No. 2, together with their aluminum or barium lakes and synonymous colorant salts, plus two further synthetic colorants declared in SPL that lack a current US certification, Coomassie Brilliant Blue G and Ponceau 4R). Secondary panels comprised propylene glycol, benzyl alcohol, aspartame (including aspartame-acesulfame), sorbitol, and sucrose.

Substances that merely contained a panel term but were chemically distinct for the relevant avoidance question, including sucrose stearate, propylene glycol alginate, and sodium starch glycolate, were excluded, with the rationale recorded in the Appendices. The wheat group deliberately captured only ingredients whose SPL name explicitly identified wheat as the botanical source: wheat starch and wheat germ. It excluded maltodextrin and source-unspecified starches, including sodium starch glycolate. This panel is a narrow screen for explicit wheat declarations and a lower-bound signal for possible gluten-related scrutiny; it does not establish gluten content or gluten-free status [5].

Outcomes

Excipient Burden

We counted distinct structured IACT ingredients declared per listing and summarized the distribution using the median, interquartile range (IQR), mean, and maximum, overall and by stratum.

Most Prevalent Declared Excipients

For every declared ingredient, we calculated the proportion of listings in the primary denominator that declared it. Recomputed counts were compared with the source census frequency table as a pipeline-consistency check.

Co-occurrence and Qualitative Excipient-Profile Diversity

We identified the most frequent pairs of co-declared excipients. A per-listing ingredient-set hash identified unique qualitative declared excipient profiles and the number of listings sharing each profile. These profiles do not establish formulation identity because ingredient quantities, manufacturing attributes, active ingredients, and strengths are not encoded in the profile definition.

Structured-Declaration Availability

We calculated the proportion of eligible listings resolving to exactly one SPL product block and, among exact matches, the proportion containing at least one structured IACT declaration. This outcome measures the availability of structured-declaration data, not the completeness or accuracy of the ingredient list.

Panel Declaration Prevalence

We calculated the listing-level declaration prevalence for each panel group overall and by-product type (prescription versus OTC), marketing category (including abbreviated new drug application {ANDA}, new drug application {NDA}/biologics license application {BLA}, authorized-generic, and OTC-monograph listings), and physical form (solid oral, liquid oral, and powder/granule). Marketing category was treated as an approval or listing pathway rather than as a synonym for brand status. The mapping of the nine source dosage-form groups to the three physical-form strata is supplied with the analysis code. The number of distinct panel-defined synthetic color additives declared per listing was exploratory.

EPC Patterns

The source census does not carry pharmacologic-class labels, so EPC labels were obtained from a second openFDA NDC Directory export, dated 2026-07-23, and joined to each cohort listing by Product NDC. The two export dates are deliberate and serve different functions: The 2026-07-18 export defined the study cohort, and the 2026-07-23 export was used only to attach class labels to listings already in that cohort. The cohort was fixed before the EPC join, and no listing was added, removed, or reclassified on the basis of the later export; a cohort listing that the later export did not label simply received no EPC and was reported in the explicit unclassified group. Using the later export therefore cannot change the primary denominator or any non-EPC result, and it attaches class labels from the export closest to the analysis date. Quantifying the effect of the five-day interval, of the 6,595 cohort listings that received no EPC, 6,579 were present in the later export but carried no EPC-annotated pharmacologic-class term, and only 16 of 50,005 listings were absent from that export altogether. The interval itself is therefore attributable to at most 16 listings, and every unlabeled listing was retained in the analytic denominator rather than excluded. A listing with multiple EPCs was counted in every applicable class; class denominators therefore overlap and cannot be summed. EPC results are unadjusted descriptions of EPC-labeled listings, not independent or causal class effects.

Within-Generic-Name Declaration Consistency

Prescription listings were grouped by normalized generic name after uppercasing and collapsing whitespace; salt forms were deliberately not merged. Eligible groups had at least two listings in the primary denominator. For each target, we first identified groups with at least one target-declaring listing. We then classified an affected group as uniformly target-declaring when every observed listing in the group declared that target. A listing that did not declare the target was not treated as confirmed target-free. Generic-name grouping did not require identical strength, dosage form, release characteristics, or therapeutic-equivalence classification and therefore did not establish pharmacy-level substitutability.

Statistical analysis

Analyses were deterministic and descriptive. Results are reported as counts and proportions for the defined snapshot census, with medians, IQRs, means, or maxima where stated. Because the analysis is a complete enumeration of the eligible snapshot rather than a probability sample, no hypothesis tests or sampling-precision intervals were used. Analysis code was written in Python (Python Software Foundation, Fredericksburg, VA) using the standard library. The manuscript was prepared with reference to STROBE guidance for cross-sectional studies [6]. The versioned code, panel definition, EPC crosswalk procedure, and computed outputs are openly archived at Zenodo [7].

Bias and sensitivity considerations

The primary analyses were complete-case descriptions of listings with at least one structured IACT declaration. The exclusion of listings without usable declarations may produce selection bias if missingness is related to excipient content; the lower structured-declaration availability among powders and granules demonstrates that missingness varies by dosage form. As a bounding check, structured declarations were absent for only 1,028 of the 51,033 exactly matched listings (97.99% availability), so reassigning every such listing to target-present or target-absent would move an overall declaration-prevalence estimate by at most roughly two percentage points; the bound is wider within powder/granule listings, where availability was 947 of 1,101 (86.01%). Listing-level estimates also weight repackaged or relabeled Product NDCs separately. The unique-profile analysis provides an alternative profile-weighted description but is not a formulation-level correction. No imputation was performed.

Ethics

This study used publicly available product-listing and labeling data and contained no human-subject or patient-level information. Institutional review board review was not applicable.

## Results

Cohort and structured-declaration availability

Of 137,124 product records in the source NDC export, 51,065 eligible distinct oral Product NDC listings entered the source census. Of these, 50,005 had an exact SPL product match and at least one structured inactive-ingredient declaration and formed the primary analytic denominator, including 38,666 prescription and 11,339 OTC listings; 38,101 ANDA and 3,140 NDA/BLA listings; and 44,963 solid oral, 4,095 liquid oral, and 947 powder/granule listings (full marketing-category breakdown in Table 1).

**Table 1:** Cohort derivation and analytic denominator, by-product type, marketing category, and physical form Percentages are of the 50,005-listing analytic denominator; the three cohort-derivation rows above that denominator are not proportions of it and are shown as "-". Product type, marketing category, and physical form are three separate, complete groupings of the same 50,005 listings: Within each grouping, the categories are mutually exclusive, and the counts sum to 50,005 (percentages to 100%, ±0.1% from rounding). "Other marketing categories" aggregates listings outside the ANDA, NDA/BLA, authorized-generic, and OTC-monograph categories NDC, National Drug Code; SPL, Structured Product Labeling; OTC, over-the-counter; ANDA, abbreviated new drug application; NDA, new drug application; BLA, biologics license application

Cohort stage/stratum	Listings (n)	Percent of analytic denominator
Source NDC product records	137,124	-
Eligible distinct oral Product NDC listings	51,065	-
With exact SPL product match	51,033	-
Analytic denominator (exact match + ≥1 structured IACT)	50,005	100.0%
Prescription (product type)	38,666	77.3%
OTC (product type)	11,339	22.7%
ANDA (marketing category)	38,101	76.2%
NDA/BLA (marketing category)	3,140	6.3%
Authorized generic (marketing category)	748	1.5%
OTC monograph (marketing category)	7,226	14.5%
Other marketing categories	790	1.6%
Solid oral (physical form)	44,963	89.9%
Liquid oral (physical form)	4,095	8.2%
Powder/granule (physical form)	947	1.9%

Of the 51,065 eligible listings, 51,033 (99.94%) resolved to exactly one SPL product block. Among those exact matches, 50,005 (97.99%) contained at least one structured IACT declaration. Structured-declaration availability was high across most strata but was lowest for oral powders and granules, among which 947 of 1,101 exact matches (86.01%) contained a structured IACT declaration. Listings without a structured declaration remained unknown and were excluded from ingredient-frequency analyses.

Overall excipient burden

Listings declared a median of nine distinct inactive ingredients (IQR, 6-12; mean, 9.48; maximum, 41). The median was nine for prescription and 10 for OTC listings, nine for ANDA and nine for NDA/BLA listings, and nine for solid versus 10 for liquid oral listings. Oral powders and granules had a median of six. The full per-listing distribution is provided with the archived outputs.

Most prevalent declared excipients

The most frequently declared excipients were magnesium stearate (68.07%), titanium dioxide (53.27%), microcrystalline cellulose (52.88%), silicon dioxide (42.09%), and corn starch (36.95%). Lactose monohydrate (30.52%) was the most prevalent single panel-relevant excipient (Table 2). Recomputed listing counts matched the source census frequency table for every reported ingredient, supporting consistency between the source and characterization pipelines.

**Table 2:** Most prevalent declared inactive ingredients among 50,005 oral drug product listings (top 20) Ingredient names are shown as declared in Structured Product Labeling

Rank	Excipient (as declared)	Listings (n)	Percent of listings
1	Magnesium stearate	34,036	68.07%
2	Titanium dioxide	26,637	53.27%
3	Cellulose, microcrystalline	26,441	52.88%
4	Silicon dioxide	21,045	42.09%
5	Starch, corn	18,477	36.95%
6	Lactose monohydrate	15,260	30.52%
7	Talc	14,194	28.39%
8	Hypromellose, unspecified	12,872	25.74%
9	Croscarmellose sodium	10,961	21.92%
10	Propylene glycol	9,980	19.96%
11	Polyethylene glycol, unspecified	9,460	18.92%
12	Gelatin, unspecified	9,184	18.37%
13	Ferrosoferric oxide	8,166	16.33%
14	Sodium starch glycolate type A potato	7,722	15.44%
15	Sodium lauryl sulfate	7,695	15.39%
16	Ferric oxide yellow	7,608	15.21%
17	Povidone, unspecified	7,308	14.61%
18	Shellac	7,047	14.09%
19	Water	7,032	14.06%
20	FD&C Blue No. 1	6,906	13.81%

Co-occurrence and qualitative excipient-profile diversity

The 50,005 listings contained 13,540 unique qualitative declared excipient profiles, a mean of 3.69 listings per profile. One profile was shared by as many as 194 listings. When each unique profile was weighted once, the median profile contained 10 ingredients. These findings describe the repetition of qualitative declared profiles and do not establish formulation identity or quantify repackager duplication.

The most frequent co-declared pairs were magnesium stearate with microcrystalline cellulose (22,076 of 50,005 listings; 44.15%), titanium dioxide with magnesium stearate (19,518; 39.03%), and magnesium stearate with silicon dioxide (16,900; 33.8%).

Panel declaration prevalence

Table 3 reports counts and declaration prevalence for every panel group. Lactose was declared in 39.34% of listings, gelatin in 18.5%, and at least one panel-defined synthetic color additive in 39.96%. Among listings declaring at least one such color additive, the mean number of distinct panel dyes was 1.82, and the maximum was eight. Soy-derived ingredients were declared in 2.83%, explicitly declared wheat ingredients in 0.01%, peanut oil in 0.14%, and sesame oil in 0.03%. For the secondary panel, propylene glycol was declared in 19.96%, sorbitol in 7.49%, sucrose in 7.89%, aspartame in 1.6%, and benzyl alcohol in 0.6%.

**Table 3:** Declared prevalence of avoidance-relevant panel excipients among 50,005 listings, overall and by stratum, reported as n (%) Each field reports the number of listings declaring the target and, in parentheses, that number as a percentage of the stratum denominator. Values are declaration prevalence. ANDA and NDA/BLA denote the marketing category. Stratum denominators are given in Table 1 Rx, prescription; OTC, over-the-counter; ANDA, abbreviated new drug application; NDA, new drug application; BLA, biologics license application

Panel group	Overall, n (%)	Rx, n (%)	OTC, n (%)	ANDA, n (%)	NDA/BLA, n (%)	Solid, n (%)	Liquid, n (%)
Lactose	19,674 (39.34%)	17,947 (46.42%)	1,727 (15.23%)	17,499 (45.93%)	1,135 (36.15%)	19,611 (43.62%)	2 (0.05%)
Gelatin	9,250 (18.5%)	7,590 (19.63%)	1,660 (14.64%)	7,307 (19.18%)	644 (20.51%)	9,249 (20.57%)	0 (0.0%)
Wheat (explicitly declared)	6 (0.01%)	0 (0.0%)	6 (0.05%)	1 (0.0%)	0 (0.0%)	4 (0.01%)	0 (0.0%)
Peanut oil	72 (0.14%)	72 (0.19%)	0 (0.0%)	70 (0.18%)	2 (0.06%)	72 (0.16%)	0 (0.0%)
Sesame oil	15 (0.03%)	15 (0.04%)	0 (0.0%)	3 (0.01%)	7 (0.22%)	14 (0.03%)	1 (0.02%)
Soy-derived	1,416 (2.83%)	1,059 (2.74%)	357 (3.15%)	1,040 (2.73%)	65 (2.07%)	1,374 (3.06%)	13 (0.32%)
Synthetic color additives	19,980 (39.96%)	13,462 (34.82%)	6,518 (57.48%)	13,683 (35.91%)	1,028 (32.74%)	17,475 (38.87%)	2,204 (53.82%)
Propylene glycol	9,980 (19.96%)	6,801 (17.59%)	3,179 (28.04%)	6,973 (18.3%)	399 (12.71%)	7,725 (17.18%)	2,237 (54.63%)
Benzyl alcohol	300 (0.6%)	118 (0.31%)	182 (1.61%)	104 (0.27%)	30 (0.96%)	105 (0.23%)	186 (4.54%)
Aspartame (phenylalanine source)	802 (1.6%)	536 (1.39%)	266 (2.35%)	626 (1.64%)	43 (1.37%)	628 (1.4%)	20 (0.49%)
Sorbitol	3,744 (7.49%)	872 (2.26%)	2,872 (25.33%)	1,134 (2.98%)	208 (6.62%)	1,554 (3.46%)	2,107 (51.45%)
Sucrose	3,944 (7.89%)	2,706 (7.0%)	1,238 (10.92%)	2,662 (6.99%)	252 (8.03%)	2,647 (5.89%)	843 (20.59%)

Declaration patterns differed across dosage forms and listing categories; stratum denominators are given in Table 1 and per-cell counts in Table 3. Lactose was declared in 19,611 of 44,963 solid listings (43.62%) and two of 4,095 liquids (0.05%), while gelatin was declared in 9,249 solids (20.57%) and zero liquids (0.0%). Both were more frequently declared in prescription than OTC listings: 17,947 of 38,666 (46.42%) versus 1,727 of 11,339 (15.23%) for lactose and 7,590 (19.63%) versus 1,660 (14.64%) for gelatin. Lactose was declared in 17,499 of 38,101 ANDA listings (45.93%) and 1,135 of 3,140 NDA/BLA listings (36.15%); the corresponding gelatin values were 7,307 (19.18%) and 644 (20.51%).

Panel-defined synthetic color additives were declared in 6,518 of 11,339 OTC listings (57.48%) and 13,462 of 38,666 prescription listings (34.82%), in 4,716 of 7,226 OTC-monograph listings (65.26%), and in 2,204 of 4,095 liquid (53.82%) versus 17,475 of 44,963 solid listings (38.87%). Propylene glycol was declared in 2,237 liquids (54.63%) and 7,725 solids (17.18%), sorbitol in 2,107 (51.45%) and 1,554 (3.46%), and benzyl alcohol in 186 (4.54%) and 105 (0.23%). Sucrose and aspartame were declared in 454 (47.94%) and 154 (16.26%) of 947 powder/granule listings, respectively.

Explicitly declared wheat ingredients appeared in six of 50,005 listings (0.01%); this count included only ingredients explicitly named as wheat-derived and did not determine gluten content. Peanut oil was declared in 72 listings (0.14%), all of which were prescription listings, and sesame oil in 15 (0.03%).

EPC patterns

The EPC join classified 43,410 of 50,005 listings (86.8%) into 338 classes. The 6,595 unclassified listings were concentrated in ANDA (3,500; 53.1%) and OTC-monograph (2,242; 34.0%) listings and were reported separately. Listings with multiple EPCs contributed to every applicable class; EPC denominators therefore overlap.

Among EPC-labeled listings, proton-pump inhibitors had a median of 18 declared ingredients and 54.4% declared gelatin. Lactose was declared in 84.4% of HMG-CoA reductase inhibitor listings. Angiotensin-II receptor blocker listings declared lactose in 57.4% and gelatin in 0.0%. Alpha-1 adrenergic agonist listings declared a panel-defined synthetic color additive in 78.4%, and H1-antihistamine listings did so in 54.6% (Table 4). These are unadjusted descriptive patterns within EPC-labeled listings.

**Table 4:** Excipient-declaration patterns by pharmacologic class (the 15 openFDA Established Pharmacologic Classes {EPCs} with the most listings; no minimum-cell threshold applied) Each excipient field reports the number of listings in that class declaring the target and, in parentheses, that number as a percentage of the class listing (n) denominator. A listing with multiple EPCs is counted under each applicable class, so class denominators overlap and cannot be summed (e.g., dextromethorphan appears under both sigma-1 agonist and NMDA-antagonist labels, hence two adjacent identical rows). EPC labels were joined from the 2026-07-23 openFDA export as described in Materials and Methods

Pharmacologic class (openFDA EPC)	Listings (n)	Median excipients	Lactose, n (%)	Gelatin, n (%)	Any dye, n (%)
Nonsteroidal anti-inflammatory drug	3,357	10	1,071 (31.9%)	653 (19.5%)	1,473 (43.9%)
Histamine-1 receptor antagonist	1,983	10	678 (34.2%)	353 (17.8%)	1,083 (54.6%)
Sigma-1 agonist	1,941	11	63 (3.2%)	325 (16.7%)	1,594 (82.1%)
Uncompetitive N-methyl-D-aspartate (NMDA) receptor antagonist	1,941	11	63 (3.2%)	325 (16.7%)	1,594 (82.1%)
Expectorant	1,429	10	87 (6.1%)	64 (4.5%)	924 (64.7%)
Beta-adrenergic blocker	1,396	10	732 (52.4%)	93 (6.7%)	481 (34.5%)
Central nervous system stimulant	1,321	10	198 (15.0%)	456 (34.5%)	773 (58.5%)
Atypical antipsychotic	1,314	9	904 (68.8%)	92 (7.0%)	315 (24.0%)
Angiotensin-II receptor blocker	1,295	9	743 (57.4%)	0 (0.0%)	163 (12.6%)
HMG-CoA reductase inhibitor	1,254	11	1,059 (84.4%)	9 (0.7%)	200 (15.9%)
Opioid agonist	1,250	8	412 (33.0%)	126 (10.1%)	557 (44.6%)
Alpha-1 adrenergic agonist	1,220	12	42 (3.4%)	186 (15.2%)	957 (78.4%)
Serotonin reuptake inhibitor	1,033	10	292 (28.3%)	218 (21.1%)	415 (40.2%)
Proton-pump inhibitor	941	18	211 (22.4%)	512 (54.4%)	436 (46.3%)
Calcium channel blocker	884	11	156 (17.6%)	287 (32.5%)	337 (38.1%)

Within-generic-name declaration consistency

The analysis included 1,207 prescription generic-name groups with at least two listings. For lactose, 657 groups had at least one declaring listing; every observed listing declared lactose in 306 of those groups (46.58%). For gelatin, every observed listing declared the target in 150 of 322 affected groups (46.58%). For panel-defined synthetic color additives, every observed listing declared at least one target color in 158 of 653 affected groups (24.2%) (Table 5).

**Table 5:** Within-generic-name declaration consistency: among prescription generic-name groups with ≥2 listings, affected groups in which every observed listing declared the target "Uniformly declaring" means every observed listing in the group declared the target; it does not establish therapeutic interchangeability, and it does not imply that nondeclaring or excluded listings are target-free. Groups are normalized generic names (salt forms not merged). "Affected groups" = groups with ≥1 declaring listing among 1,207 eligible groups

Target excipient	Affected groups (≥1 declaring listing)	Groups uniformly declaring	Percent uniformly declaring
Lactose	657	306	46.58%
Gelatin	322	150	46.58%
Soy-derived	94	15	15.96%
Synthetic color additives	653	158	24.2%
Wheat (explicitly declared)	0	0	-
Peanut oil	2	1	50.0%
Sesame oil	1	0	0.0%
Propylene glycol	455	98	21.54%
Sorbitol	138	24	17.39%
Sucrose	200	44	22.0%
Benzyl alcohol	25	5	20.0%
Aspartame (phenylalanine source)	41	4	9.76%

Uniformly declaring groups with large numbers of listings included buspirone hydrochloride (264 listings), rosuvastatin calcium (201), carvedilol (170), simvastatin (163), and warfarin sodium (83) for lactose. For gelatin, every observed listing declared the target for benzonatate (132 listings), atomoxetine (109), clindamycin hydrochloride (90), and progesterone (67). For panel-defined synthetic color additives, every observed listing declared a target for hydralazine hydrochloride (132), clindamycin hydrochloride (90), and hydroxyzine pamoate (82).

All 67 observed progesterone listings declared gelatin and peanut oil. All 94 isotretinoin listings declared a soy-derived ingredient. For soy-derived ingredients overall, every observed listing declared the target in 15 of 94 affected groups (15.96%). Among secondary-panel targets, every observed listing declared the target in 21.54% of affected propylene glycol groups (98 of 455 groups), 24 of 138 (17.39%) for sorbitol, 44 of 200 (22.0%) for sucrose, five of 25 (20.0%) for benzyl alcohol, and four of 41 (9.76%) for aspartame. These findings establish uniform structured declarations within the analyzed groups; they do not establish therapeutic interchangeability or the absence of a target from nondeclaring or excluded listings.

## Discussion

Principal findings

This census provides a current, reproducible description of inactive-ingredient declarations in a US oral drug-listing snapshot. Listings declared a median of nine inactive ingredients, and magnesium stearate, titanium dioxide, and microcrystalline cellulose each appeared in roughly half or more of listings. The 50,005 listings contained 13,540 unique qualitative excipient profiles, demonstrating the extensive repetition of excipient profiles across listings without establishing that listings sharing a profile are the same formulation.

Declaration patterns differed across EPCs. HMG-CoA reductase inhibitor listings frequently declared lactose, proton-pump inhibitor listings had high ingredient counts and gelatin declarations, and alpha-1 adrenergic agonist and H1-antihistamine listings frequently declared panel-defined synthetic colors. These are class-level descriptions rather than effects attributable to pharmacologic class; dosage-form and listing-category composition may contribute to the patterns.

Lactose and panel-defined synthetic color additives were each declared in roughly two in five complete-case listings, while gelatin was declared in nearly one in five. These proportions describe listings with usable structured declarations, not patient exposure, dispensing volume, ingredient quantity, or reaction risk.

The within-name analysis identified affected prescription generic-name groups in which every observed listing declared a target. Such groups contained no listing in the analytic dataset whose structured declaration omitted that target. The magnitude of that pattern depends on how a group is composed: It is most pronounced in small groups and attenuates substantially in groups with 10 or more listings or with more than one distinguishable formulation record, and the sensitivity analyses in the Appendices should be read alongside the headline figures. The pattern persists under every restriction tested, which is the finding of interest; its size is not a single number. Conversely, a listing that omitted a target declaration was only a candidate for further verification, not evidence of target absence. Because normalized generic-name groups did not enforce strength, dosage form, release characteristics, or therapeutic-equivalence criteria, the analysis should not be interpreted as identifying products that a pharmacist can substitute directly.

Comparison with prior work

Reker et al. established the broad prevalence and complexity of inactive ingredients in US oral solid medications using Pillbox data [1]. They also analyzed unique inactive-ingredient combinations and the availability of formulations avoiding allergy-associated ingredients at the active-ingredient level. The present findings are directionally concordant with that work, including the frequent declaration of lactose in solid oral products.

This study extends rather than introduces those concepts. It uses a 2026 openFDA NDC and DailyMed SPL snapshot, applies an exact-product-match rule, includes liquid and powder/granule oral products, uses explicit UNII-defined target panels, describes structured-declaration availability, and reports EPC-stratified patterns. It also publishes a deterministic source census and characterization workflow designed for independent audit. The resulting contribution is a current public-data replication and extension, not evidence that the underlying clinical problem is newly discovered.

Methodological contribution and public-data quality

The study separates three related but distinct units: Product NDC listings, structured ingredient declarations, and unique qualitative excipient profiles. Product NDC listings represent labeler-submitted records and include repackagers and relabelers. Structured declarations describe what an exactly matched SPL record affirms. Unique profiles identify identical qualitative sets but contain no quantity or manufacturing information. Keeping these units separate prevents repeated profiles from being mislabeled as confirmed formulations, and the within-name sensitivity analysis shows why that separation matters in practice.

Among exact SPL product matches, 50,005 of 51,033 (97.99%) contained at least one structured IACT declaration. This permits large-scale description within records containing structured data, but it does not validate the completeness of any ingredient list. Availability was also uneven, reaching 947 of 1,101 (86.01%) for powders and granules. An automated product-level query that treats a missing declaration as evidence of absence will therefore return a falsely reassuring answer at a measurable rate, and that rate is highest in the dosage form most used in pediatric practice. Such tools must preserve an explicit unknown state rather than inferring absence.

The source files were checksum-verified, targets were defined through explicit UNII sets, the extraction pipeline passed 14 automated checks and 18 of 18 independent manual spot checks, and analyses were deterministic. The manuscript-specific code, panel version, EPC crosswalk, and reported outputs were archived together before submission [7].

Clinical interpretation

Structured-declaration data can be used to prioritize product-level verification. When every observed listing in a generic-name group declares a target, the dataset does not identify a same-name listing with an omitting structured declaration. When at least one listing omits the target, that listing may warrant a review of the current full label and, when clinically necessary, confirmation with the labeler or manufacturer.

Any product change must also satisfy pharmaceutical and legal substitution requirements. FDA therapeutic-equivalence evaluations consider active ingredient, strength, dosage form, route, labeling, and bioequivalence; products sharing a generic name are not necessarily interchangeable [8]. Actual wholesaler or pharmacy inventory is outside the NDC Directory and was not measured.

Declared presence should not be equated with contraindication. Structured declarations provide no ingredient quantity, and refinement can substantially reduce residual allergenic protein in soybean and peanut oils [9,10]. Soy lecithin and oils of uncertain refinement may have different residual-protein profiles. Accordingly, a soy- or peanut-derived declaration identifies a product for scrutiny in an allergic patient rather than establishing clinical reaction risk. The converse also applies: the absence of a structured declaration is not proof of absence.

A qualitative declaration is also not an interchangeable clinical information for a second reason: some declared excipients are not inert with respect to drug absorption. Polyol sweeteners such as sorbitol can accelerate small-intestinal transit and, in sufficient quantity, reduce the systemic exposure of co-administered drugs, and surfactant and polymer excipients can alter solubility and intestinal transport. This is relevant to interpretation because sorbitol was declared in 51.45% of liquid oral listings in this census. However, structured declarations carry no quantity, so this analysis cannot estimate any excipient-related effect on bioavailability and is not offered as evidence of one; the census identifies which listings declare which excipients, which is an input to that question rather than an answer to it.

Regulatory context

The FASTER Act added sesame as the ninth major food allergen for packaged-food labeling, including dietary supplements, but those food-allergen labeling requirements do not create an equivalent "Contains" declaration for drug products [11]. In this census, sesame oil was identified through the drug's inactive-ingredient declaration. The absence of an allergen summary statement on drug products means that, for sesame, peanut, and soy-derived ingredients, the structured inactive-ingredient declaration is the only machine-readable signal available to a patient or a consumer-facing tool. The coverage figures reported here therefore describe the practical ceiling on what any such tool can currently answer.

FDA revoked authorization for FD&C Red No. 3 in food and ingested drugs, with a January 2028 compliance date for ingested drug products [12]. The panel-wide synthetic-color estimate of 19,980 of 50,005 listings (39.96%) combines 32 colorant UNIIs and must not be read as the proportion of listings affected by that revocation; the colorant-specific estimate reported below is smaller by a factor of roughly 24.

Implications for policy and practice

Three findings bear on decisions outside the dataset.

The FD&C Red No. 3 transition is narrow, and its reformulation burden is concentrated. The revocation applies to a specific colorant, and this census can size it directly rather than by inference: 827 of 50,005 listings (1.65%) declared FD&C Red No. 3 or its erythrosine salt, representing 4.14% of the 19,980 listings declaring any panel-defined synthetic color. Those 827 listings map to 229 distinct labelers, 141 normalized generic names, and 287 distinct declared excipient profiles, which is the more informative measure of reformulation work because listings sharing a profile are candidates for a shared reformulation. Declaration was higher in prescription than OTC listings (1.88% versus 0.87%) and, notably, highest in oral powders and granules (4.22%) and lowest in liquids (0.17%) (Appendices). A regulatory-impact estimate built from the panel-wide 39.96% figure would overstate the affected population by more than an order of magnitude.

The within-name view adds a supply consideration. Among the 119 prescription generic-name groups containing at least one Red No. 3-declaring listing, only six (5.04%) had every observed listing declare it; in the remaining 113 (94.96%), at least one same-name listing already did not declare the colorant. At the level of the generic name, therefore, the catalog already contains nondeclaring alternatives for the large majority of affected products, and the transition is unlikely to create broad name-level availability gaps. The six exceptions, which include meclofenamate sodium, flurazepam hydrochloride, opicapone, and nicardipine, are small groups in which no observed listing omits the colorant and are the candidates for monitoring before the compliance date. This is a declaration-level signal about a listing snapshot, not a supply forecast: it does not measure inventory, dispensing volume, manufacturer reformulation plans, or products entering or leaving the market before 2028.

Structured-declaration completeness is itself a data-policy target. Availability was 97.99% overall but 86.01% among oral powders and granules, which is the form most often used in pediatric and enteral-tube administration. The practical consequence is asymmetric: a tool that reports "not present" when the record is merely silent produces a false negative precisely where the underlying record is weakest. Two corrections follow, and neither requires new authority. Systems that consume Structured Product Labeling should distinguish "no declaration present" from "ingredient absent" and surface that distinction to the user. Also, because availability varies by dosage form rather than at random, completeness is measurable per form and could be monitored as a labeling data-quality metric rather than assumed.

Excipient avoidance interacts with generic substitution in a way substitution law does not address. State substitution frameworks and FDA therapeutic-equivalence ratings turn on active ingredient, strength, dosage form, route, labeling, and bioequivalence; excipient content is not among the criteria [8]. Yet, in a substantial minority of affected prescription generic-name groups, 29.02% of groups with 10 or more listings for lactose and higher in smaller groups (Appendices), every observed listing declared the target. For a patient avoiding that excipient, substitution within the generic name is therefore not a reliable remedy, and the dataset offers no same-name listing whose structured declaration omits it. That is a practical argument for excipient information to be visible at the point of dispensing rather than reachable only through the full label, and it is the concrete use we intend for these outputs: prioritizing which products need verification, not replacing verification.

## Conclusions

Inactive-ingredient declarations are common across US oral Product NDC listings and vary across dosage-form, listing-category, and EPC strata. Many listings share the same qualitative excipient profile, although profile identity does not establish formulation identity. Within some affected prescription generic-name groups, every observed listing declared the target excipient, a pattern that persists under sensitivity analysis but attenuates in larger and more heterogeneous groups. Public structured-label data can support population-level surveillance and identify products for verification, but they do not establish ingredient absence, patient exposure, current inventory, or therapeutic interchangeability, and automated tools built on them must return an explicit unknown state where no structured declaration exists.

## Appendices

Table 6 shows the avoidance-relevant excipient panel.

**Table 6:** Avoidance-relevant excipient panel: FDA Unique Ingredient Identifier (UNII) sets with inclusion/exclusion rationale "Include = yes" rows define each avoidance-relevant panel group. "Include = no" rows are namesake or related substances deliberately excluded, with the reason given. Panel version 1, archived at Zenodo (DOI: 10.5281/zenodo.21515694) MW, molecular weight; SPL, Structured Product Labeling; PKU, phenylketonuria; GI, gastrointestinal

Group	Tier	UNII	Preferred name	Include	Rationale
Lactose	Primary	EWQ57Q8I5X	Lactose monohydrate	Yes	Dietary lactose; lactose intolerance/galactosemia avoidance
Lactose	Primary	3SY5LH9PMK	Anhydrous lactose	Yes	Dietary lactose
Lactose	Primary	J2B2A4N98G	Lactose, unspecified form	Yes	Dietary lactose
Gelatin	Primary	2G86QN327L	Gelatin, unspecified	Yes	Animal-derived; vegetarian/vegan/kosher/halal avoidance
Gelatin	Primary	1T8387508X	Gelatin type B bovine (160 bloom)	Yes	Bovine gelatin
Gelatin	Primary	WIL1404U79	Gelatin type B bovine (230 bloom)	Yes	Bovine gelatin
Gelatin	Primary	6EG4DCS5TJ	Gelatin type A porcine (195 bloom)	Yes	Porcine gelatin
Gelatin	Primary	A7JR5F8DLH	Gelatin type B bovine (200 bloom)	Yes	Bovine gelatin
Gelatin	Primary	F5AJW0ONK4	Gelatin type B bovine (150 bloom)	Yes	Bovine gelatin
Gelatin	Primary	7Z075S9991	Gelatin type A porcine (160 bloom)	Yes	Porcine gelatin
Gelatin	Primary	AHQ60JKI5D	Marine non-gelling gelatin, high MW	Yes	Fish gelatin (also fish-allergy-relevant)
Wheat (explicitly declared)	Primary	79QS2MG2LP	Starch, wheat	Yes	Explicitly wheat-derived starch; gluten-risk flag; celiac avoidance
Wheat (explicitly declared)	Primary	YR3G369F5A	Wheat germ	Yes	Explicitly wheat-derived; gluten-risk flag
Peanut oil	Primary	5TL50QU0W4	Peanut oil	Yes	Peanut allergy (refined-oil risk debated; presence still labeled)
Sesame oil	Primary	QX10HYY4QV	Sesame oil	Yes	Sesame allergy (FASTER Act allergen)
Soy-derived	Primary	1DI56QDM62	Lecithin, soybean	Yes	Soy-derived (highly refined lecithin usually low allergen risk, discuss)
Soy-derived	Primary	241ATL177A	Soybean oil	Yes	Soy-derived (refined oil usually low allergen risk, discuss)
Soy-derived	Primary	A2M91M918C	Hydrogenated soybean oil	Yes	Soy-derived
Soy-derived	Primary	H1109Z9J4N	Hydrogenated soybean lecithin	Yes	Soy-derived
Soy-derived	Primary	L7HT8F1ZOD	Soybean	Yes	Soy-derived
Soy-derived	Primary	1T6N4D9YV6	Soybean phosphatidylcholine	Yes	Soy-derived
Soy-derived	Primary	HBA528N3PW	Soy acid	Yes	Soy-derived
Soy-derived	Primary	P491F5HP1U	Soy sphingolipids	Yes	Soy-derived
Soy-derived	Primary	CQD833204Z	Lysophosphatidylcholine, soybean	Yes	Soy-derived
Soy-derived	Primary	NWB9514AZM	Soy amino acids	Yes	Soy-derived
Soy-derived	Primary	R44IWB3RN5	Soy protein	Yes	Soy protein (highest allergen relevance in group)
Synthetic color additives	Primary	H3R47K3TBD	FD&C Blue No. 1	Yes	Certified synthetic dye
Synthetic color additives	Primary	WZB9127XOA	FD&C Red No. 40	Yes	Certified synthetic azo dye
Synthetic color additives	Primary	H77VEI93A8	FD&C Yellow No. 6	Yes	Certified synthetic azo dye (Sunset Yellow)
Synthetic color additives	Primary	35SW5USQ3G	D&C Yellow No. 10	Yes	Certified synthetic dye
Synthetic color additives	Primary	L06K8R7DQK	FD&C Blue No. 2	Yes	Certified synthetic dye (indigotine)
Synthetic color additives	Primary	J9EQA3S2JM	FD&C Blue No. 1 aluminum lake	Yes	Lake form of certified dye
Synthetic color additives	Primary	CQ3XH3DET6	D&C Yellow No. 10 aluminum lake	Yes	Lake form
Synthetic color additives	Primary	767IP0Y5NH	D&C Red No. 28	Yes	Certified synthetic dye
Synthetic color additives	Primary	4AQJ3LG584	FD&C Blue No. 2 aluminum lake	Yes	Lake form
Synthetic color additives	Primary	PN2ZH5LOQY	FD&C Red No. 3	Yes	Certified synthetic dye (erythrosine; current FDA action)
Synthetic color additives	Primary	9DBA0SBB0L	D&C Red No. 33	Yes	Certified synthetic azo dye
Synthetic color additives	Primary	3P3ONR6O1S	FD&C Green No. 3	Yes	Certified synthetic dye
Synthetic color additives	Primary	2LRS185U6K	D&C Red No. 27	Yes	Certified synthetic dye
Synthetic color additives	Primary	2S42T2808B	D&C Red No. 30	Yes	Certified synthetic dye
Synthetic color additives	Primary	GYP6Z2JR6Q	FD&C Yellow No. 6 aluminum lake	Yes	Lake form
Synthetic color additives	Primary	I753WB2F1M	FD&C Yellow No. 5	Yes	Tartrazine, classic sensitivity dye
Synthetic color additives	Primary	ZK64F7XSTX	D&C Red No. 27 aluminum lake	Yes	Lake form
Synthetic color additives	Primary	6T47AS764T	FD&C Red No. 40 aluminum lake	Yes	Lake form
Synthetic color additives	Primary	GE75M6YV5W	D&C Red No. 30 aluminum lake	Yes	Lake form
Synthetic color additives	Primary	1678RKX8RT	D&C Red No. 22	Yes	Certified synthetic dye
Synthetic color additives	Primary	D3741U8K7L	Indigotindisulfonate sodium	Yes	Indigo carmine, same colorant as FD&C Blue No. 2
Synthetic color additives	Primary	ECW0LZ41X8	D&C Red No. 7	Yes	Certified synthetic dye
Synthetic color additives	Primary	JQ6BLH9FR7	FD&C Yellow No. 5 aluminum lake	Yes	Tartrazine lake
Synthetic color additives	Primary	X3W0AM1JLX	FD&C Red No. 4	Yes	Certified synthetic dye
Synthetic color additives	Primary	K4XZD9W99K	D&C Red No. 6 barium lake	Yes	Lake form
Synthetic color additives	Primary	481744AI4O	D&C Red No. 6	Yes	Certified synthetic dye
Synthetic color additives	Primary	L3FT3LUT29	D&C Red No. 33 aluminum lake	Yes	Lake form
Synthetic color additives	Primary	WJE3T5596E	FD&C Red No. 4 free acid	Yes	Certified synthetic dye (free acid)
Synthetic color additives	Primary	4XYU5U00C4	D&C Black No. 2	Yes	Certified carbon-black colorant (D&C designation); included as a declared synthetic color additive
Synthetic color additives	Primary	8TL7LH93FM	Erythrosine sodium anhydrous	Yes	Erythrosine salt (FD&C Red No. 3 colorant)
Synthetic color additives	Primary	M1ZRX790SI	Brilliant Blue G	Yes	Coomassie-type synthetic dye; lacks a current US certification but declared in SPL; included as a synthetic color additive (n = 1)
Synthetic color additives	Primary	Z525CBK9PG	Ponceau 4R	Yes	Synthetic azo dye (E124); lacks a current US certification but declared in SPL; included as a synthetic color additive (n = 1)
Propylene glycol (PG)	Secondary	6DC9Q167V3	Propylene glycol	Yes	Intolerance/toxicity interest; plain PG only
Benzyl alcohol	Secondary	LKG8494WBH	Benzyl alcohol	Yes	Preservative sensitivity interest
Aspartame (phenylalanine source)	Secondary	Z0H242BBR1	Aspartame	Yes	PKU-relevant phenylalanine source
Aspartame (phenylalanine source)	Secondary	IFE6C6BS24	Aspartame acesulfame	Yes	Contains aspartame moiety, PKU-relevant
Sorbitol	Secondary	506T60A25R	Sorbitol	Yes	Sugar alcohol; GI intolerance/hereditary fructose intolerance
Sorbitol	Secondary	8KW3E207O2	Sorbitol solution	Yes	Sorbitol in solution
Sorbitol	Secondary	9E0S3UM200	Noncrystallizing sorbitol solution	Yes	Sorbitol in solution
Sucrose	Secondary	C151H8M554	Sucrose	Yes	Dietary sugar; diabetes/fructose interest; plain sucrose only
Excluded with rationale	Excluded	Z135WT9208	Carbomer homopolymer type B (allyl sucrose crosslinked)	No	Crosslinker residue, not dietary sucrose
Excluded with rationale	Excluded	274KW0O50M	Sucrose stearate	No	Sucrose ester surfactant, not dietary sucrose
Excluded with rationale	Excluded	26CD3J2R0C	Propylene glycol alginate	No	Distinct substance from plain PG
Excluded with rationale	Excluded	658271J00C	Sorbitol monooleate	No	Ester surfactant, not free sorbitol
Excluded with rationale	Excluded	H8AV0SQX4D	Sodium starch glycolate (SSG) type A	No	Excluded from wheat/gluten: pharmaceutical-grade sodium starch glycolate is potato- or corn-derived, and gluten is negligible
Excluded with rationale	Excluded	SP4S77AHO6	Sodium starch glycolate type B	No	Excluded from wheat/gluten: same rationale as SSG type A
